# Ivabradine Ameliorates Kidney Fibrosis in L-NAME-Induced Hypertension

**DOI:** 10.3389/fmed.2020.00325

**Published:** 2020-07-10

**Authors:** Peter Stanko, Tomas Baka, Kristina Repova, Silvia Aziriova, Kristina Krajcirovicova, Andrej Barta, Pavol Janega, Michaela Adamcova, Ludovit Paulis, Fedor Simko

**Affiliations:** ^1^Institute of Pathophysiology, Faculty of Medicine, Comenius University, Bratislava, Slovakia; ^2^Institute of Normal and Pathological Physiology, Centre of Experimental Medicine, Slovak Academy of Sciences, Bratislava, Slovakia; ^3^Institute of Pathological Anatomy, Faculty of Medicine, Comenius University, Bratislava, Slovakia; ^4^Department of Physiology, School of Medicine, Charles University, Prague, Czechia; ^5^3rd Department of Internal Medicine, Faculty of Medicine, Comenius University, Bratislava, Slovakia; ^6^Institute of Experimental Endocrinology, Biomedical Research Center, Slovak Academy of Sciences, Bratislava, Slovakia

**Keywords:** ivabradine, L-NAME, hypertension, fibrosis, nephroprotection

## Abstract

Hypertension-induced renal injury is characterized by structural kidney alterations and function deterioration. Therapeutics for kidney protection are limited, thus novel renoprotectives in hypertension are being continuously sought out. Ivabradine, an inhibitor of the I_f_ current in the sinoatrial node reducing heart rate (HR), was shown to be of benefit in various cardiovascular pathologies. Yet, data regarding potential renoprotection by ivabradine in hypertension are sparse. Thirty-six adult male Wistar rats were divided into non-diseased controls and rats with N^G^-nitro-L-arginine methyl ester (L-NAME)-induced hypertension to assess ivabradine's site-specific effect on kidney fibrosis. After 4 weeks of treatment, L-NAME increased the average systolic blood pressure (SBP) (by 27%), decreased glomerular density (by 28%) and increased glomerular tuft area (by 44%). Moreover, L-NAME induced glomerular, tubulointerstitial, and vascular/perivascular fibrosis by enhancing type I collagen volume (16-, 19- and 25-fold, respectively). L-NAME also increased the glomerular type IV collagen volume and the tubular injury score (3- and 8-fold, respectively). Ivabradine decreased average SBP and HR (by 8 and 12%, respectively), increased glomerular density (by 57%) and reduced glomerular tuft area (by 30%). Importantly, ivabradine decreased type I collagen volume at all three of the investigated sites (by 33, 38, and 72%, respectively) and enhanced vascular/perivascular type III collagen volume (by 67%). Furthermore, ivabradine decreased the glomerular type IV collagen volume and the tubular injury score (by 63 and 34%, respectively). We conclude that ivabradine attenuated the alterations of glomerular density and tuft area and modified renal fibrosis in a site-specific manner in L-NAME-hypertension. It is suggested that ivabradine may be renoprotective in hypertensive kidney disease.

## Introduction

Chronic kidney disease (CKD), determined by a decline of total glomerular filtration rate and albuminuria persisting >3 months, is a severe health and social problem. CKD afflicts almost 15% of the global population and significantly worsens life expectancy (1). Although a considerable number of etiologic factors may come into force, diabetes mellitus, and arterial hypertension clearly dominate (2). CKD in hypertension is characterized by a persistent systemic blood pressure overload gradually exceeding auto-regulatory mechanisms for maintaining adequate glomerular filtration pressure. Glomerular hypertension and hyperfiltration are associated with compensatory glomerular hypertrophy, albuminuria, and persistent inflammation (3). Progressive necrotic or apoptotic cell death is followed by glomerulosclerosis, tubular atrophy, and tubulointerstitial fibrosis representing the principle pathologic components and therapeutic targets of CKD (4).

Ivabradine, the heart rate (HR)-reducing selective inhibitor of the sinoatrial I_f_ current, was proved to attenuate morbidity in heart failure (5). The HR-reducing effect of ivabradine was declared the principle mechanism of its therapeutic benefit. However, several of ivabradine's pleiotropic effects have recently emerged, which suggests the possibility of using ivabradine in yet off-label indications such as endothelial dysfunction (6), hypertensive heart disease (7), and hypertension with elevated or non-dipping HR (8, 9).

This study aimed to show whether ivabradine is able to modify kidney alterations in N^G^-nitro-L-arginine methyl ester (L-NAME)-induced hypertension. We investigated ivabradine's potential antifibrotic effect in a site-specific manner as glomerulosclerosis (glomerular fibrosis), tubulointerstitial fibrosis, and arteriosclerosis (vascular fibrosis) with perivascular fibrosis.

## Materials and Methods

Thirty-six 3-month-old male Wistar rats (Department of Toxicology and Laboratory Animals Breeding, Slovak Academy of Sciences, Dobra Voda, Slovakia) were divided into 4 groups (*n* = 9 animals per group) and treated for 4 weeks as follows: control (C; untreated), ivabradine (Iva; 10 mg/kg/day; Servier, Suresnes, France), L-NAME (LN; 40 mg/kg/day; Sigma-Aldrich Chemie, Munich, Germany) and L-NAME plus ivabradine in corresponding doses (LN+Iva). Both L-NAME and ivabradine were dissolved in drinking water and their concentrations were adjusted to daily water consumption to ensure the correct dosage. The rats were individually housed and maintained under standard laboratory conditions (12:12-h light-dark cycle at 22 ± 2°C temperature and 55 ± 10% humidity) with free access to food and water. The study was conducted in accordance with the Guide for the Care and Use of Laboratory Animals published by the US National Institutes of Health (NIH Publication No. 85-23, revised 1996). The protocol was approved by the ethical committee of the Institute of Pathophysiology, Faculty of Medicine, Comenius University, Bratislava, Slovakia (approval number: 1306/14-221).

Systolic blood pressure (SBP) and heart rate (HR) were measured once a week in each animal by non-invasive tail-cuff plethysmography (Hugo-Sachs Elektronic, Freiburg, Germany). After 4 weeks of treatment, the rats were euthanized by isoflurane inhalation and left kidneys were used for subsequent histopathological analysis. The kidney samples were fixed in 4% formaldehyde for 24 h, embedded in paraffin and cut in 5 μm sections. Three sets of deparaffinized and rehydrated sections were stained with: (i) hematoxylin-eosin (H-E) for glomerular morphometry and tubular injury scoring; (ii) picrosirius red (PSR; 0.1% sirius red F3BA in a saturated water solution of picric acid for 90 min and washed in 0.01 N HCl for 2 min) for a quantitative analysis of kidney fibrosis; and (iii) type IV collagen immunostaining (anti-collagen IV antibody; ab6586; Abcam, Cambridge, UK was used for immunostaining conforming the manufacturer's protocol: a heat-mediated antigen retrieval was followed by overnight incubation with primary anti-collagen IV antibody at 4°C; a horseradish peroxidase-conjugated secondary anti-rabbit IgG antibody with a 3,3′-diaminobenzidine chromogen and hematoxylin counterstain was used for visualization; ab205718; Abcam, Cambridge, UK) to determine type IV collagen volume in glomeruli. Histopathological observations were performed using transmitted or polarized light microscopy on a NIKON Eclipse Ti C2+ microscope (NIKON, Tokyo, Japan). The rendered images were analyzed by NIKON NIS-Elements Analysis software (NIKON, Tokyo, Japan) and ImageJ version 1.52p for Windows (National Institutes of Health, Bethesda, MD, USA). All histopathological analyses were performed by an experienced examiner blinded to the group identity.

For glomerular morphometry, H-E-stained sections were analyzed at 10x magnification using transmitted light microscopy and NIKON NIS-Elements Analysis software as follows: (i) to assess glomerular numerical density per area, well-preserved glomeruli were counted in a digital frame of 1 mm^2^ placed over the kidney cortex in 10 microscopic fields per animal (i.e., 90 per group; *n* = 9 animals per group); (ii) to assess glomerular tuft area, perpendicular maximum and minimum diameters (d_max_ and d_min_, respectively) of 10 random glomerular tufts per animal (i.e., 90 per group; *n* = 9 animals per group) were measured to subsequently calculate tuft ellipse areas by using the formula: glomerular tuft area = π(d_max_/2)(d_min_/2) (10, 11).

In order to obtain a quantitative analysis of kidney fibrosis, PSR-stained sections were analyzed at 100x magnification using polarized light microscopy and ImageJ software as follows: PSR increases birefringence of collagen fibers type-dependently, thus visualizing thick type I collagen (Col-I, 1.6-2.4 μm in diameter) in red-orange shades and thin type III collagen (Col-III, <0.8 μm in diameter) in green-yellow shades; by setting the appropriate “hue” thresholds of the color spectrum, the red-orange and green-yellow shaded areas were expressed as the percentage of the total area of interest (AOI) by ImageJ processing. To particularly detail kidney fibrosis, three AOIs were determined by employing a method described previously (12): (i) to assess glomerular fibrosis, 50 AOIs per animal (i.e., 450 per group; *n* = 9 animals per group) of 50 × 50 μm each, placed at intraglomerular space were examined (Figure 2A1); (ii) to assess tubulointerstitial fibrosis, 50 AOIs per animal (i.e., 450 per group; *n* = 9 animals per group) of 72 × 192 μm each, placed at interstitial cortex space including no glomeruli or vessels were examined (Figure 2B1); (iii) to assess vascular/perivascular fibrosis, 5 AOIs per animal (i.e., 45 per group; *n* = 9 animals per group) were selected by the tight-cropping of an artery between 50 and 100 μm in diameter (corresponding to interlobar, arcuate, and interlobular arteries) in each; only cross-sectionally captured arteries were considered (Figure 2C1).

In order to determine type IV collagen (Col-IV) volume in glomeruli, anti-Col-IV-immunostained sections were analyzed at 200x magnification using transmitted light microscopy and ImageJ software as follows (13): the anti-Col-IV-immunostain visualizes Col-IV in brown shades (Figure 3A); by setting the appropriate “hue” threshold of the color spectrum, the brown shaded area was expressed as the percentage of the total glomerular AOI by ImageJ processing. Ten glomerular AOIs per animal (i.e., 90 per group; *n* = 9 animals per group) of 50 × 50 μm each, placed at intraglomerular space were examined.

Tubular injury was determined as tubular injury score by employing a method described previously (14). Briefly, 20 cortical fields per animal (i.e., 180 per group; *n* = 9 animals per group) in H-E-stained sections were analyzed at 100x magnification using transmitted light microscopy. Tubular injury was defined as tubular dilatation, atrophy, cast formation, sloughing of tubular epithelial cells, or thickening of the tubular basement membrane (Figure 4A). The tubular injury was semi-quantitatively scored using the following scoring system: Score 0, no tubular injury; Score 1, <10% of tubules injured; Score 2, 10–25% of tubules injured; Score 3, 26–50% of tubules injured; Score 4, 51–75% of tubules injured; Score 5, >75% of tubules injured.

The results are presented as the mean ± SEM. A Shapiro-Wilk normality test was used to determine data distribution. The one-way two-tailed analysis of variance (ANOVA) followed by a Holm-Sidak *post-hoc* test was used for statistical analysis. A Spearman correlation was used to analyze the relationship between glomerular tuft area and glomerular Col-IV volume, and tubular injury score and tubulointerstitial fibrosis. Statistical significance was defined as *P* < 0.05. The statistical analysis was conducted using GraphPad Prism 8 for Windows (GraphPad Software, La Jolla, CA, USA).

## Results

**Figure 1:**
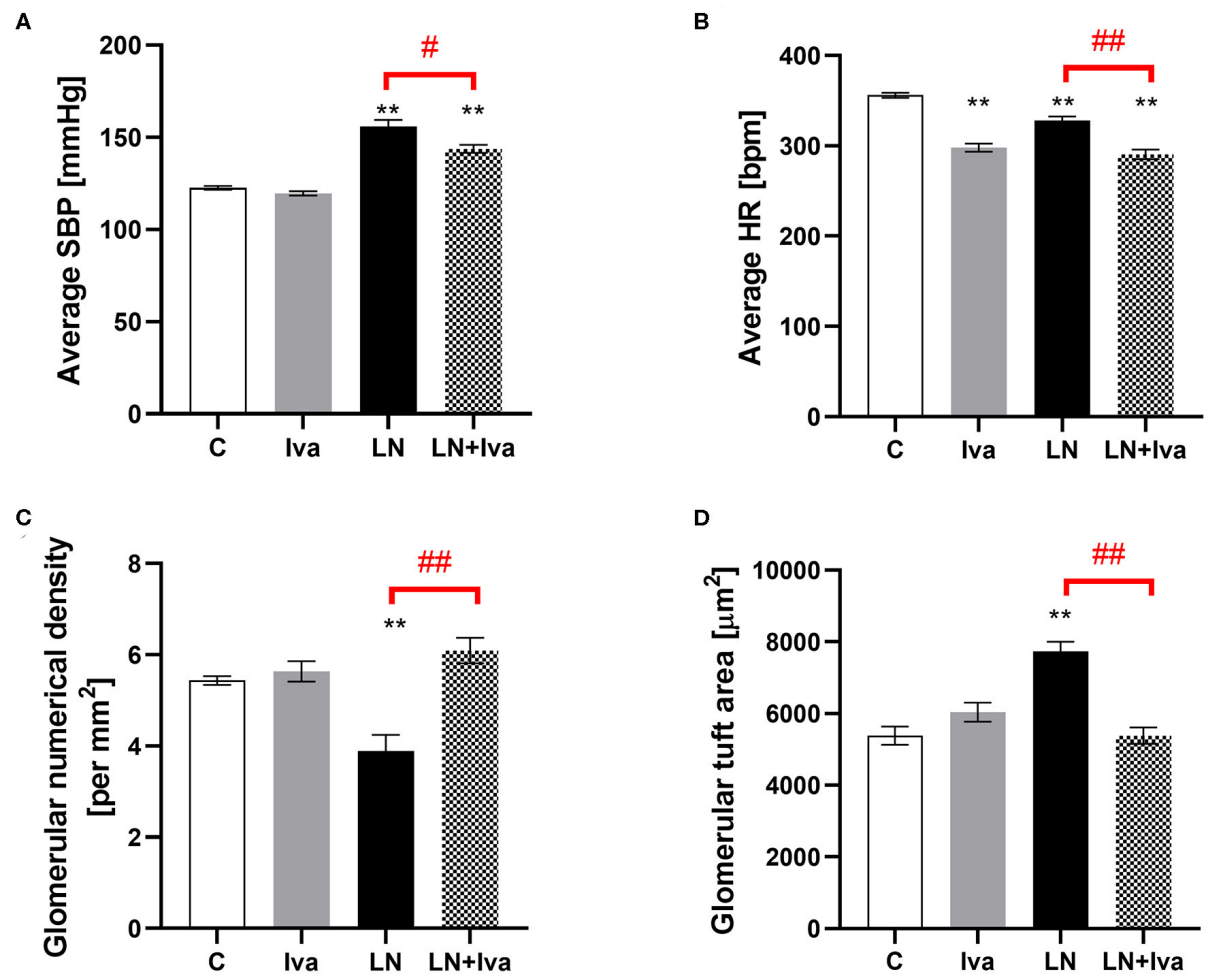
Effect of ivabradine on average systolic blood pressure (SBP) **(A)**, average heart rate (HR) **(B)**, glomerular numerical density per area **(C)**, and glomerular tuft area **(D)** in L-NAME-induced hypertension after 4 weeks of treatment. C, controls; Iva, ivabradine; LN, L-NAME; *n* = 9 animals per group. One-way two-tailed ANOVA followed by Holm–Sidak *post-hoc test*; **P < 0.01 vs. C; ^#^*P* < 0.05 vs. LN; ^*##*^*P* < 0.01 vs. LN.

The SBP and HR averaged over the 4-week-course of treatment were 122.6 ± 1.05 mmHg and 356.0 ± 2.75 bpm in controls; L-NAME increased (*P* < 0.01) average SBP by 27% and decreased (*P* < 0.01) average HR by 8%. In the L-NAME group, ivabradine decreased both average SBP and HR by 8% (*P* < 0.05) and 12% (*P* < 0.01), respectively; in controls, ivabradine decreased (*P* < 0.01) average HR by 16% and had no effect on average SBP (Figures 1A,B).

The glomerular numerical density per area was 5.43 ± 0.1 per mm^2^ in controls and L-NAME decreased (*P* < 0.01) it by 28%. In the L-NAME group, ivabradine increased (*P* < 0.01) the glomerular numerical density per area by 57% (Figure 1C). The glomerular tuft area was 5,383 ± 256 μm^2^ in controls and L-NAME increased (*P* < 0.01) it by 44%. In the L-NAME group, ivabradine decreased (*P* < 0.01) the glomerular tuft area by 30% (Figure 1D).

**Figure 2:**
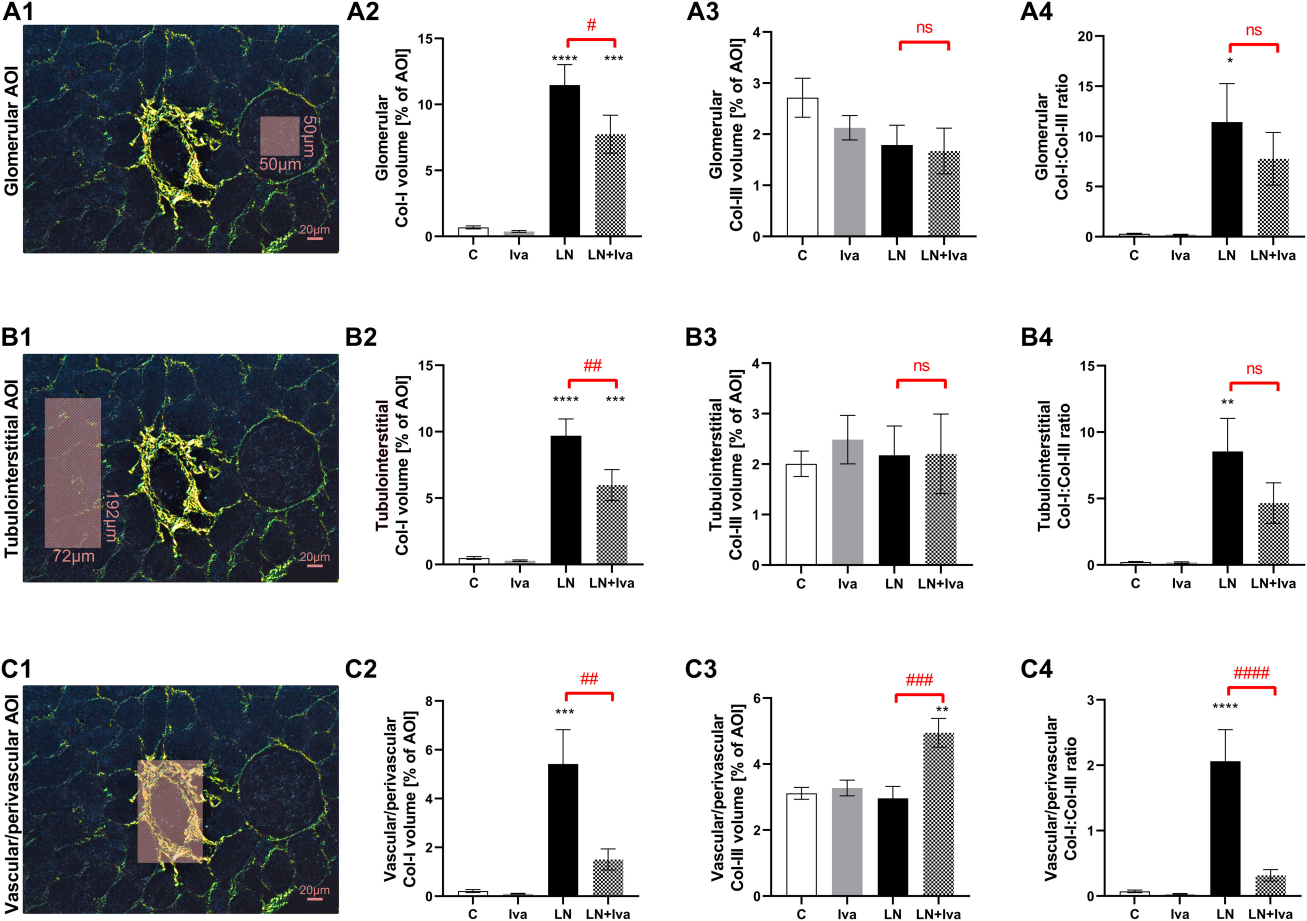
Effect of ivabradine on kidney fibrosis detailed as glomerular **(A)**, tubulointerstitial **(B)**, and vascular/perivascular fibrosis **(C)** in L-NAME-induced hypertension after 4 weeks of treatment. PSR-stained sections at 100x magnification using polarized light microscopy. AOI, area of interest depicted as shaded rectangle; C, controls; Col-I, type I collagen; Col-III, type III collagen; Iva, ivabradine; LN, L-NAME; *n* = 9 animals per group. One-way two-tailed ANOVA followed by Holm–Sidak *post-hoc test*; *P < 0.05 vs. C; **P < 0.01 vs. C; ***P < 0.001 vs. C; ****P < 0.0001 vs. C; ^#^*P* < 0.05 vs. LN; ^*##*^*P* < 0.01 vs. LN; ^*###*^*P* < 0.001 vs. LN; ^*####*^*P* < 0.0001 vs. LN; ns, non-significant.

Quantitative analysis of glomerular fibrosis: in controls, the volume of Col-I and Col-III in intraglomerular AOI were 0.68 ± 0.11 and 2.71 ± 0.38%, respectively; L-NAME increased (*P* < 0.0001) the proportion of Col-I by 1,584%. In the L-NAME group, ivabradine decreased (*P* < 0.05) the proportion of Col-I by 33%. The Col-I:Col-III ratio was 0.29 ± 0.06 in controls and L-NAME increased (*P* < 0.05) it by 3,880%; ivabradine had no effect on the ratio (Figure 2A).

Quantitative analysis of tubulointerstitial fibrosis: in controls, the volume of Col-I and Col-III in tubulointerstitial AOI were 0.49 ± 0.11 and 2.01 ± 0.25%, respectively; L-NAME increased (*P* < 0.0001) the proportion of Col-I by 1,894%. In the L-NAME group, ivabradine decreased (*P* < 0.01) the proportion of Col-I by 38%. The Col-I:Col-III ratio was 0.22 ± 0.04 in controls and L-NAME increased (*P* < 0.01) it by 3,734%; ivabradine had no effect on the ratio (Figure 2B).

Quantitative analysis of vascular/perivascular fibrosis: in controls, the volume of Col-I and Col-III in vascular/perivascular AOI were 0.21 ± 0.06 and 3.11 ± 0.18%, respectively; L-NAME increased (*P* < 0.001) the proportion of Col-I by 2,487%. In the L-NAME group, ivabradine decreased (*P* < 0.01) the proportion of Col-I by 72% and increased (*P* < 0.001) the proportion of Col-III by 67%. The Col-I:Col-III ratio was 0.07 ± 0.02 in controls and L-NAME increased (*P* < 0.0001) it by 2,796%; ivabradine decreased (*P* < 0.0001) the ratio by 85% (Figure 2C).

**Figure 3:**
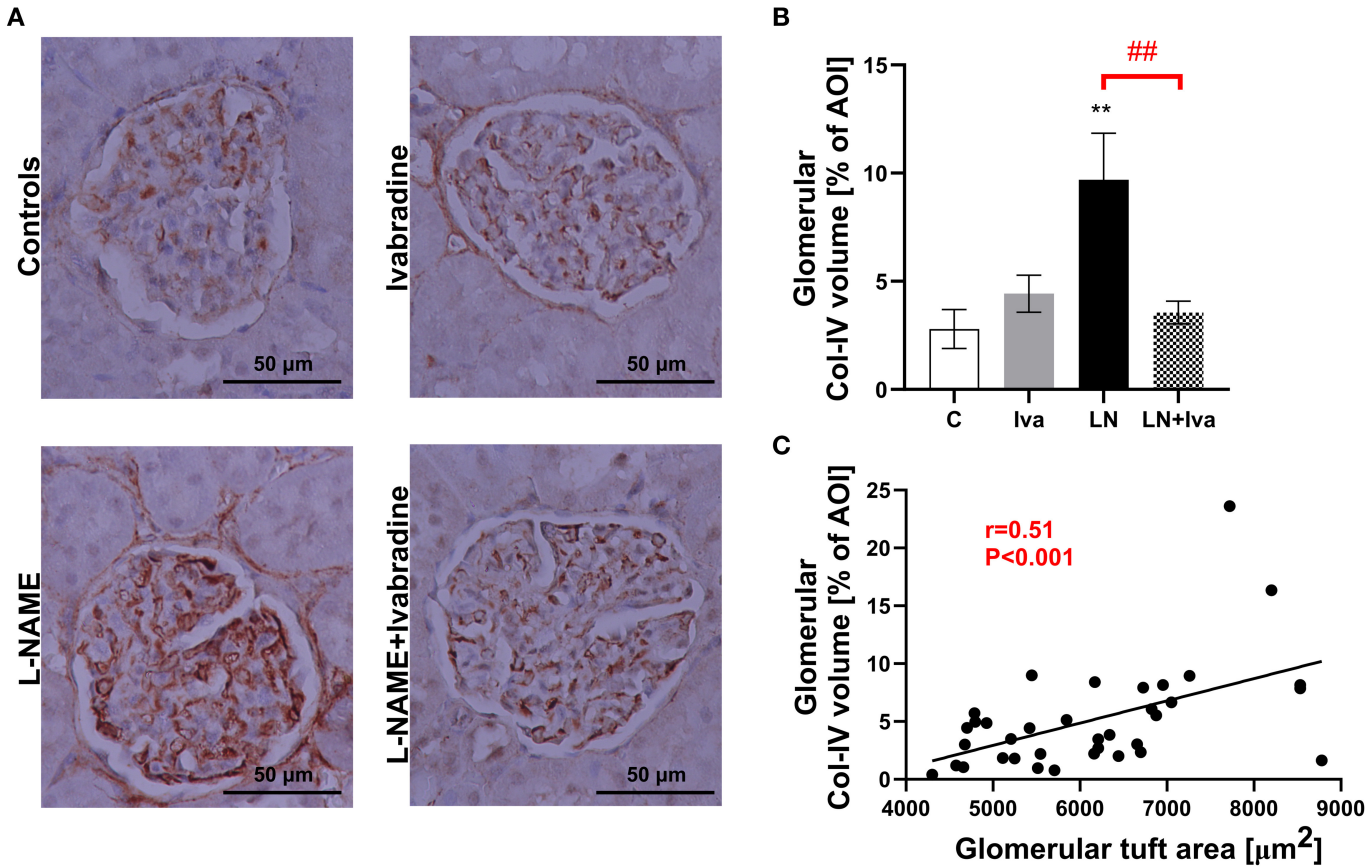
Effect of ivabradine on type IV collagen volume in glomeruli **(A,B)** and the relationship between glomerular tuft area and glomerular type IV collagen volume **(C)** in L-NAME-induced hypertension after 4 weeks of treatment. For **(A)**: anti-collagen IV-immunostained sections at 200x magnification using transmitted light microscopy. For **(B)**: C, controls; Iva, ivabradine; LN, L-NAME; *n* = 9 animals per group. One-way two-tailed ANOVA followed by Holm–Sidak *post-hoc test*; **P < 0.01 vs. C; ^*##*^*P* < 0.01 vs. LN. For **(C)**: AOI, area of interest; Col-IV, type IV collagen. Spearman correlation; *n* = 9 animals per group.

The volume of Col-IV in glomerular AOI was 2.80 ± 0.90% in controls and L-NAME increased (*P* < 0.01) it by 245%. In the L-NAME group, ivabradine decreased (*P* < 0.01) glomerular Col-IV volume by 63% (Figures 3A,B). The glomerular Col-IV volume significantly (*P* < 0.001) correlated with glomerular tuft area (Spearman *r* = 0.51) (Figure 3C).

**Figure 4:**
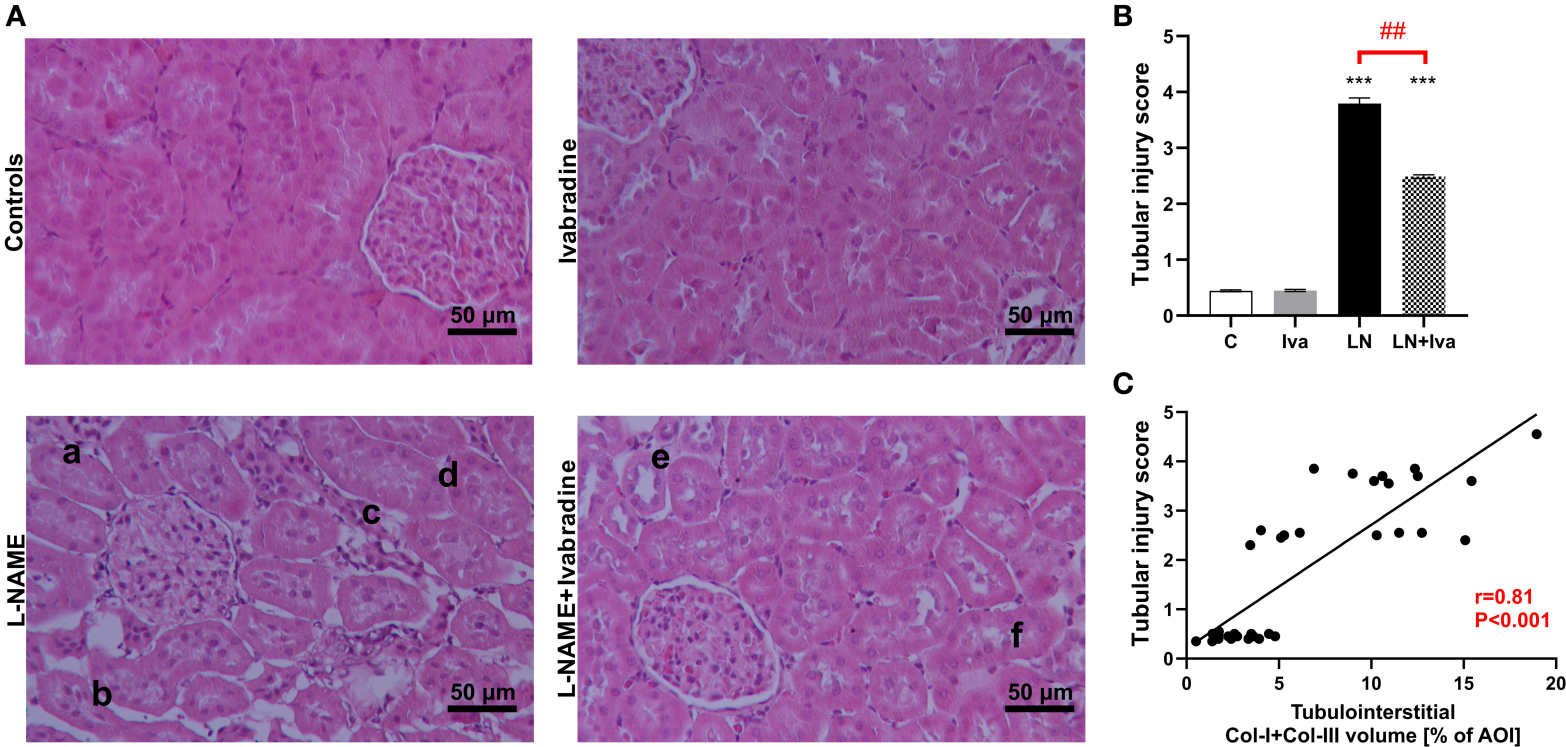
Effect of ivabradine on tubular injury **(A,B)** and the relationship between tubular injury score and tubulointerstitial fibrosis **(C)** in L-NAME-induced hypertension after 4 weeks of treatment. For **(A)**: H-E-stained sections at 100x magnification using transmitted light microscopy; a, tubular cast; b, tubular atrophy and interstitial thickening; c, interstitial cellular infiltration; d, tubular basal membrane thickening; e, tubular dilatation; f, tubular cell sloughing. For **(B)**: C, controls; Iva, ivabradine; LN, L-NAME; *n* = 9 animals per group. One-way two-tailed ANOVA followed by Holm–Sidak *post*-*hoc* test; ***P < 0.001 vs. C; ^*##*^*P* < 0.01 vs. LN. For **(C)**: AOI, area of interest; Col-I, type I collagen; Col-III, type III collagen. Spearman correlation; *n* = 9 animals per group.

The tubular injury score was 0.45 ± 0.02 in controls and L-NAME increased (*P* < 0.001) it by 754%. In the L-NAME group, ivabradine decreased (*P* < 0.01) the tubular injury score by 34% (Figures 4A,B). The tubular injury score significantly (*P* < 0.001) correlated with the sum of Col-I and Col-III volume in tubulointerstitial AOI (Spearman *r* = 0.81) (Figure 4C).

## Discussion

The increased SBP, induced by a 4-week L-NAME administration, was associated with decreased glomerular density, increased glomerular tuft area, tubular injury, and profound renal fibrosis. Besides reducing the average HR and SBP, ivabradine increased glomerular density and reduced glomerular tuft area. Furthermore, ivabradine ameliorated the L-NAME-induced kidney fibrosis site-dependently: it decreased Col-I volume in glomerular, interstitial, and vascular/perivascular fibrosis and increased Col-III volume in vascular/perivascular fibrosis. Ivabradine also mitigated the increase of both the glomerular Col-IV volume and the tubular injury score in L-NAME-hypertension. To the best of our knowledge, this is the first study analysing L-NAME-induced kidney fibrosis and renoprotection by ivabradine in a site-specific manner.

L-NAME inhibits nitric oxide (NO) synthase activity and decreases cyclic guanosine monophosphate (cGMP) concentration shown in various tissues including heart, aorta, brain, and kidney, thus resulting in NO-deficient hypertension (15–19). NO's waning vasodilative and antiproliferative effects associated with concomitant neurohumoral activation (20) were shown to result in target organ damage (7, 18, 19). In kidneys, L-NAME-hypertension induces glomerulosclerosis, tubulointerstitial fibrosis, and tubular atrophy associated with a deteriorated glomerular filtration rate and increased urinary protein excretion (21–23); these alterations are administered by a cluster of pro-inflammatory and pro-proliferative hormones, cytokines and growth factors (24).

The renal antifibrotic effect of ivabradine observed in this study was similar to previous findings within the heart: ivabradine reduced cardiac collagen and improved left ventricular function in post-myocardial infarction (MI) rats (25) and cholesterol-fed rabbits (26). Antiremodeling by ivabradine may be associated with some of its pleiotropic effects. Indeed, ivabradine improved endothelial function in ApoE knockout mice by reducing vascular oxidative stress and preventing endothelial NO synthase uncoupling (27, 28) and in patients with coronary artery disease after complete revascularisation (6). Increased NO-availability by various interventions was previously shown to reduce the L-NAME's proliferative effect not only in the heart and aorta, but also in the brain and kidneys (17, 18, 28). Besides improving NO-availability, ivabradine was shown to reduce serum angiotensin II in both post-MI rats (25) and hypercholesterolemic rabbits (26), and to decrease serum aldosterone in L-NAME-induced hypertension (7). The potential inhibition of the renin-angiotensin-aldosterone system might contribute to the antiremodeling effect of ivabradine. Indeed, previous data in L-NAME-hypertension indicated an angiotensin converting enzyme inhibitor to mitigate remodeling of the heart, aorta (19, 20, 29–31) and kidneys (21). Furthermore, one of the principle factors of CKD management is the reduction of hemodynamic overload. The recommended target SBP values in hypertensive patients with CKD are below 130 vs. 140 mmHg in hypertensive patients without kidney disease (32). Presumably, HR-reduction by ivabradine may be renoprotective via diminishing the hemodynamic burden by both the rate-pressure working product decline and vascular shear stress modulation (33). Here, in line with our previous experiments (7, 8, 34), ivabradine reduced both the average SBP and HR, which indeed might have contributed to the renoprotection. The HR reduction by L-NAME found in this study is consistent with previous results in L-NAME-hypertension by our laboratory (7, 8, 15) and others (35, 36). Several plausible mechanisms of heart rate reduction in L-NAME-hypertension were suggested, including the baroreceptor-mediated modulation of the autonomic nervous system (37, 38) and the direct effect of NO-deficiency on cardiac function (39, 40). Yet, ivabradine is an open-channel I_f_-blocker, i.e., the ivabradine molecule is able to access its binding site in the f-channel only when the channel is open. This can underlie ivabradine's use-dependence, i.e., a blocking action that is more pronounced the more frequently the f-channel is open, implying that the higher the HR the larger the HR-reducing effect of ivabradine (41, 42). This might explain our finding that ivabradine reduced HR in controls by 16%, but only 12% in L-NAME-hypertension, since the HR in L-NAME-hypertension was already decreased by L-NAME below the values seen in controls.

Col-I and Col-III are the most abundant collagen types in the extracellular matrix (43, 44). In kidneys, they were found co-expressed at all three of the investigated sites, i.e., glomeruli, tubulointerstitium and vasculature (45), and gradually deposited from the early stages of kidney fibrosis (45, 46). Col-I and Col-III co-expression is considered to provide a tissue with high tensile strength, but also contribute to its extensile properties (44, 46). Indeed, Col-I exerts high tensile strength and its expression is associated with tissue stiffness, whilst Col-III is more distensible and its expression refers to tissue elasticity, distensibility, and softness (43, 44). Thus, a high Col-I:Col-III ratio was found in tissues with high mechanical stiffness and low elasticity such as bones, and low Col-I:Col-III ratio was found in tissues with high elasticity, distensibility, or softness such as lung, bladder, and blood vessels (44). In cardiovascular remodeling, the Col-I:Col-III ratio is considered a marker of tissue stiffness determining mechanical properties and was shown to be associated with adverse outcomes (47). Indeed, an elevated Col-I:Col-III ratio was associated with increased myocardial stiffness and electrical instability of the myocardium (48), and increased stiffness of vessels including the aorta and arteries (49, 50). Increased stiffness of the remodeled vasculature was shown to be prognostically unfavorable in hypertension (51). We previously found an up-ward shift of the Col-I:Col-III ratio in a remodeled heart (52) and aorta (49) in a model of continuous light-induced hypertension. Reducing the Col-I:Col-III ratio in vessels was associated with improved hemodynamics in continuous light-induced hypertension (49) and pulmonary arterial hypertension (53). In this study, we dosed L-NAME for only 4 weeks (reaching a moderate increase in 4-week average systolic blood pressure) to assess early hypertensive kidney damage and its potential reversibility with ivabradine. Although Col-I and Col-III are deposited from early stages of kidney fibrosis (45, 46), NO deficiency in L-NAME-hypertension was found to specifically up-regulate collagen I expression in kidneys at an early stage even preceding the increase in blood pressure (54). This might explain the increased Col-I expression (early activation) and unchanged Col-III expression (activation lagging) observed in early hypertensive kidney damage in this study. Furthermore, in L-NAME-treated rats, ivabradine increased Col-III volume solely in the vascular/perivascular fibrosis while a profound drop in Col-I volume prevailed at all three of the investigated sites. Previously, ivabradine was shown to increase aortic compliance in apolipoprotein E-deficient mice (55), improve carotid pulsatile arterial hemodynamics in spontaneously hypertensive rats (56), restore acetylcholine-induced maximal dilatation of renal and cerebral arteries in dyslipidaemic mice (57), and most importantly, improve myocardial perfusion in post-MI rats by ameliorating perivascular fibrosis in small resistant coronary arteries (25). Therefore, by virtue of Col-III's elastic properties, the vascular/perivascular Col-III enhancement associated with the reduction of the Col-I:Col-III ratio by ivabradine observed in our study implies improved arterial compliance and pulsatile hemodynamics (49).

Col-IV, a main component of the glomerular basement membrane, is considered to play a critical role in glomerular pathology (58). Indeed, capillary expansion and mesangial cell stretching by increased intraglomerular pressure, often seen in hypertension, were found to provoke increased mesangial extracellular matrix (including Col-IV) production and deposition (59). Therefore, increased Col-IV protein expression was found in kidneys in various animal models of hypertension such as spontaneously hypertensive rats (60), angiotensin II-induced hypertension (61) or 2 kidneys, 1 clip model of hypertension (62), and also in patients with preeclampsia or other hypertensive syndromes in pregnancy (63). In L-NAME-hypertension, in particular, an exaggerated Col-IV gene and protein expression within the renal vasculature associated with glomerulosclerosis was found (64). Nonetheless, to the best of our knowledge, this is the first study determining Col-IV volume specifically in glomeruli in L-NAME-hypertension, where ivabradine mitigated the L-NAME-induced increase of glomerular Col-IV volume, thus supporting ivabradine's beneficial effect on glomerulosclerosis in L-NAME-hypertension.

Furthermore, in this study, L-NAME-hypertension induced tubular injury that correlated with tubulointerstitial fibrosis. This is in line with findings from other animal models of hypertension such as spontaneously hypertensive rats (65), angiotensin II-induced hypertension (66), and the Dahl salt-sensitive rat model of hypertension (67). Yet, mechanisms underlying tubular injury in hypertension are puzzling. Indeed, there were suggested (i) hemodynamics-dependent mechanisms, including tubular atrophy following glomerulotubular disconnection associated with glomerular injury (68), and (ii) hemodynamics-independent mechanisms, including renal oxidative stress and inflammation (69). In this study, ivabradine mitigated tubular injury and decreased tubulointerstitial fibrosis in L-NAME-hypertension, which were presumably associated with ivabradine's effects on both hemodynamics-dependent and independent mechanisms of tubular injury.

Recently, the plasma and urinary markers of Col-III (70), Col-IV and Col-VI (43) turnover have been shown to be a proxy for kidney fibrosis correlating with kidney function deterioration, and severity and the prognosis of CKD, thus holding promise as a novel, non-invasive diagnostic and prognostic tool to monitor kidney fibrosis in CKD (70, 71). This histopathological study was designed to directly and site-specifically determine collagen volumes in L-NAME-induced kidney fibrosis. It may be of interest to correlate histopathology and plasma or urinary markers of kidney fibrosis and function. Yet, this was beyond the scope and possibilities of the present histopathological study.

We conclude that ivabradine mitigated alterations to glomerular density and tuft area and site-specifically modified renal fibrosis in L-NAME-hypertension. These results suggest that ivabradine may be renoprotective in hypertensive kidney disease.

## Data Availability Statement

The datasets generated for this study are available on request to the corresponding author.

## Ethics Statement

The animal study was reviewed and approved by The Ethical Committee of the Institute of Pathophysiology, Faculty of Medicine, Comenius University, Bratislava, Slovakia.

## Author Contributions

FS and TB conceived and designed the study and drafted the manuscript. PS, TB, KR, SA, and KK carried out animal experiments. PS, AB, PJ, MA, and LP conducted histopathological analysis. All authors participated in data analysis and interpretation, manuscript revision, and approved the submitted version.

## Conflict of Interest

The authors declare that the research was conducted in the absence of any commercial or financial relationships that could be construed as a potential conflict of interest.
